# Effects of hexavalent chromium on the biology of *Steinernema feltiae*: evaluating sublethal endpoints for ecotoxicity testing

**DOI:** 10.1371/journal.pone.0320329

**Published:** 2025-04-01

**Authors:** Anique Godjo, Darren Mc Donald, Lucile Ansaldi, Islam A. A. Darwish, John L. Byrne, Thomais Kakouli-Duarte

**Affiliations:** Department of Applied Science, Molecular Ecology and Nematode Research Group, enviroCORE, South East Technological University, Carlow, Ireland; University of Limpopo, SOUTH AFRICA

## Abstract

Essential information about the effects of a pollutant on an ecosystem can be obtained by observing how it influences a bioindicator organism. Hexavalent chromium (Cr VI^+^) naturally occurs in Irish agricultural soils at levels of up to 250 mg/kg, which raises concerns about potential negative impacts on human health and the surrounding areas. This research aimed to assess the sublethal effect concentrations (up to 300 ppm) of Cr VI^ +^ on the entomopathogenic nematode (EPN) *Steinernema feltiae* focusing on endpoints such as nematode movement and host finding ability in contaminated sand and pathogenicity, percentage penetration, sex ratio and reproduction in *Galleria mellonella*. To achieve that, an Irish isolate of *S. feltiae* [strain SB 12 (1)], was used in all experiments. The attraction of nematodes to the insect host was tested using PVC tubes of various lengths, containing sand with various concentrations of Cr VI^ +^ (50-300 ppm in increments of 50). The replication was tenfold and the insect mortality was recorded at the end of the experiment. Results showed that there was a significant effect of Cr VI^ +^ on the pathogenicity, movement and host finding ability of the nematodes in contaminated sand, and on the percentage of penetration in an insect host. However, no significant effects among the studied Cr VI^ +^ concentrations were observed in *S. feltiae* reproduction in *G. mellonella.* Similarly, the presence of the toxicant (at low concentration of 12ppm) did not affect the growth of the nematode symbiotic bacteria in liquid and solid media (TSA and NBTA). Reproduction, unlike the other sublethal parameters tested, appeared not to be an optimal endpoint for assessing soil Cr VI^ +^ risk contamination. Overall, this study confirms the excellent potential of *S. feltiae* to be used as a suitable sentinel organism in assessing the risk of Cr VI^ +^ soil contamination especially in the contexts of agriculture and soil health.

## 1. Introduction

Increasing global anthropogenic activity results in a corresponding increase in environmental pollution. This disturbance in the environment can be caused by a wide range of compounds including heavy metals. These pollutants can alter ecosystem functions and the overall quality of a habitat [1]. In Europe and across the globe, heavy metal pollution in soil occupies an important place in the debate about their implications for food safety and food security [2]. Chromium is a relatively abundant element on earth and can exist in multiple oxidative forms starting from -2 to + 6 [3]. Hexavalent chromium (Cr VI^+^) is among the most stable forms of the element that can be found in natural environmental conditions [4]. Cr VI^ +^ is mobile and can be bioaccumulated in animal cells and tissues through biological membranes. Therefore, Cr VI^ +^ is listed among the dangerous elements for the environment and represents an important threat to human, animal and environmental health. A long term exposure to Cr VI^ +^ may lead to several nasal disorders including irritations of the respiratory tract which may result in lung and nasal sinus cancer [5]. As Cr VI^ +^ is ubiquitous in soils naturally, its large amounts in soil occur as a result of human industrial practices. Chromium is quite abundant in most soils in the European Union (EU) and high concentrations have been noted in some EU regions putting at risk millions of hectares of agricultural land on the continent [2]. In Ireland, Cr VI^ +^ occurs naturally in agricultural soils at the rate of 5-250mg/kg [6]. A number of approaches have been used to define risk levels associated with heavy metal presence in the soil internationally [7]. Therefore, thresholds and guideline values for metal concentrations in soils have been defined to identify soil contamination and the need for remediation. In the EU, these values have been discussed for chromium presence in soil and the threshold and guideline values are 100mg/Kg and 200-300mg/kg, respectively [2].

Using a whole organism to risk assess soil contamination with a pollutant is an important component of an ecotoxicological analysis approach. Soil fauna constitute an important part of the terrestrial ecosystem. Strongly associated via the soil food web, members of soil fauna play an important role in the ecosystem and any disturbance due to pollution can result in the alteration of the food web and soil health [8]. Therefore, members of soil fauna are better suited to risk assess soil quality and the effect of pollutants on ecosystems. Nematodes are the most abundant soil species-rich and by number of individuals metazoans on earth [9]. They are ubiquitous organisms and are present in all habitats including freshwater, marine sediments and soils [10] and represent in that sense the best candidate phylum to evaluate the effect of a pollutant on the terrestrial ecosystem. Several studies including recent ones conducted by scientists in the field confirmed the fact that nematodes offer extremely sensitive and responsive measurements as bioindicators of environmental heavy metal contamination [11,12]. This approach has been used in previous studies and especially by the German company Ecossa which developed the nematode *Caenorhabditis elegans* as a bioindicator to risk assess soil pollution [1]. Remarkable outputs have been reported leading to an establishment of an International Standardised (ISO) protocol for pollutant laboratory testing [1]. The use of more than one eucaryotic species can increase predictability in toxicology tests [13]. In that respect, in order to provide more alternatives in predictive ecotoxicology studies, there is a need to investigate additional nematode species possessing the prerequisite characteristics to act as suitable bioindicators. Entomopathogenic nematodes (EPN) are soil inhabiting, easy to cultivate, ubiquitous in soil and demonstrated some resistance to low concentrations of toxicants including Cr VI^ +^ [14]. An Irish isolate of *Steinernema feltiae*, has been investigated for its differential gene expression upon Cr VI^ +^ exposure and some sublethal parameters [14–16]. These studies yielded promising results and gave hope for the use of *S. feltiae* as a sentinel organism for soil Cr VI^ +^ contaminated risk assessment.

The present research aimed to investigate, in laboratory conditions, the sublethal endpoints that could be used to risk assess Cr VI^ +^ soil contamination using *S. feltiae* as a sentinel. Specific objectives were to study the effect of Cr VI^ +^ soil pollution on *S. feltiae* attraction to *Galleria mellonella*, the nematode ability to kill an insect host, the nematode penetration percentage, the nematode sex ratio within hosts and the nematode reproduction with *G. mellonella*.

## 2. Materials and methods

### 2.1 Source of nematode and insect host

An Irish isolate of *Steinernema feltiae* [strain SB 12 (1)] was used in this study. This strain has been previously isolated from Irish soil in a past enviroCORE project [14,17] and is currently maintained through subsequent in *vivo* cultures in the laboratory using *G. mellonella* (wax worm) larvae. Late instar larvae of *G. mellonella* were supplied by a local pet shop (Davy’s Pets, Strawhall Ind EST., Carlow) and used as insect host in all bioassays. Freshly harvested nematode infective juveniles (IJ) were used in this study and kept at 10°C when not used immediately.

### 2.2 Effect of Cr VI^ +^ on nematode pathogenicity to an insect host

Sterile 24 multi-well plates were used in this experiment. In each well, 3g of sterile (121 °C, 15 psi, 1h) play sand was placed after removal of bigger particles by sieving with a N 40 mesh. The sand in each well was spiked with Cr VI^ +^ (sodium dichromate Cr2 Na2 O7 2H_2_O) with concentrations (Cr VI^ +^ to sand) varying from 50 to 300ppm (with increments of 50ppm). The appropriate amount of Cr VI^ +^ was diluted in 100 µl of sterile distilled water and added to the sand in each well. Twenty IJ were added to each well in 100 µl of distilled water. The moisture level of the sand was adjusted to 10% in each well by adding an additional 100 µl of sterile distilled water. Two untreated controls were applied including a positive control (CT+) which received no Cr VI^ +^ but accepted nematode suspension and a negative control (CT-) which received no Cr VI^ +^ and no nematodes. A third treated control (CT) was applied and received Cr VI^ +^ at 300 ppm but no nematodes. The whole experiment was repeated twice under the same conditions but at a different time period to ensure reproducibility of the results. Each 24 multi-well plate represented one replicate, and each treatment was replicated three times for the first run of experiment. In the second run of experiment, the number of replicates per treatment was increased to five, making the total number of replicates per treatment in both experiments to eight. Plates were incubated in the dark at room temperature (20° C) to let the nematodes equilibrate in the new Cr VI^ +^ contaminated environment. Twenty-four hours later, one fresh *G. mellonella* larva (257.16 ±55 g) was placed in each well, and plates were sealed with parafilm to avoid moisture loss and prevent larvae from escaping. Sealed plates were thereafter incubated in the dark at room temperature and subsequently, insect death was recorded in each treatment three days later.

### 2.3 Effect of Cr VI^ +^ on *S. feltiae* penetration percentage into an insect host and sex ratio

Six dead insects were randomly selected from each treatment from the pathogenicity experiment above (2.2) except the negative controls where no nematodes were applied. Insect cadavers were rinsed thoroughly with sterile distilled water and placed individually in a Petri dish (6 cm diameter). The petri dishes were lined with Whatman filter paper (Cat No 1002090) and incubated in the dark at room temperature (20 °C) for 2 days to allow the penetrated IJ to develop to adults. After incubation, each insect cadaver was gently dissected in distilled water under a stereoscope to record the number of nematodes (males and females) that developed inside the insect host.

### 2.4 Effect of Cr VI^ +^ on the reproduction potential of *S. feltiae*

An additional six dead insects per treatment, except in the negative controls. were randomly selected from the pathogenicity experiment (section 2.2 above), weighed and placed individually on White traps [18]. The White trap containers were incubated at room temperature in the dark to evaluate the effect of Cr VI^ +^ on the number of emerged IJ from each insect cadaver. The date on which the first IJ was observed in a container was recorded to evaluate the effect of Cr VI^ +^ on the developmental period of *S. feltiae*. Infective juveniles were collected in each container until no nematodes could be observed.

### 2.5 Effect of Cr VI ^+^ on the growth of the symbiotic bacteria of the nematodes

*Xenorhabdus bovienii* is the bacterial symbiont associated with *S. feltiae* [19,20]. In this research, this bacterium was isolated from the haemolymph of *G. mellonella* larvae infected with freshly harvested IJ of *S. feltiae* using the method previously described [21]. To serve as a positive control, *Xenorhabdus nematophilus* which is the symbiotic bacterium associated with the nematode *Steinernema carpocapsae* [22], was included in this experiment. Both bacterial cells were grown on Nutrient Bromothymol blue Agar (NBTA) plates in the dark for 48 h at 28°C [23] and once a pure colony of the bacterium was obtained, it was grown on Tryptic Soy Agar (TSA) in the same condition for experimental use (S1 File). The effect of Cr VI^ +^ on the development of the symbiotic bacteria was assessed both on solid (TSA and NBTA) and liquid (Nutrient Broth, NB) media. On solid media, the bacteria were grown overnight in Tryptic Soy Broth (TSB) at 28°C on a shaker in the dark. At 0.7 nm optical density (OD_600_), measured with the Shimadzu UV-1800 120V UV/Vis Spectrophotometer, 50μl of the bacterial suspension was evenly plated on TSA and NBTA in five replicates for each bacterium. Subsequently, five drops (10μl each) of filter-sterilised Cr VI^ +^ at 300 ppm were added to the plates on different spots (S1 File) and allowed to dry in the laminar flow for 2 min. Thereafter, the plates were incubated upside down at 28°C in the dark to allow bacterial growth. Forty-eight hours later, the development of the symbiotic bacteria was assessed. In liquid medium, Falcon tubes were filled with 30 ml of NB. Replication was five-fold. Afterward, 1 ml of bacterial suspension (0.7nm OD_600)_ was transferred from TSB to the NB in each tube. Cr VI^ +^ was dissolved in distilled water, filter sterilised (0.22 μm mesh) and added to the medium to adjust the concentration to 300 ppm and 12 ppm. Untreated (control) tubes did not receive any Cr VI^ +^ treatment. The tubes were sealed and incubated on a shaker at 28°C in the dark. Bacterial growth was assessed by measuring the OD_600_ after 02, 04, 06, 08, 24 and 48 hours in all tubes. Both experiments were repeated twice using different fresh bacterial cultures.

### 2.6 Nematode attraction to the host in Cr VI^ +^ contaminated sand

The ability of *S. feltiae* to move, locate and kill the insect host in Cr VI^** +**^ contaminated sand was investigated in this study. Polyvinyl chloride (PVC) tubing (4 cm diameter) were cut into different lengths (5 cm, 10 cm, 15 cm and 20 cm) and filled with sterile (121°C, 1h) play sand. The sand was spiked with Cr VI^ +^ dissolved in distilled water to have a final concentration (Cr VI^ +^ to sand) of 50-300 ppm (in increments of 50 ppm) and adjusted at 10% moisture. Infective juveniles of *S. feltiae* were initially exposed to Cr VI^ +^ at 50-300 ppm, dissolved in distilled water, for 24 hours and thereafter rinsed three times with sterile distilled water to remove any excess chemical. One hundred alive Cr VI^ +^ exposed IJ were added to each PVC tubing at the top end in a 100 μl distilled water suspension. A single late instar *G. mellonella* larva (257.16 ±55 g) was placed at the opposite end, and both PVC openings were sealed with parafilm to avoid water evaporation, placed vertically in a completely randomised design and incubated at room temperature at 20 °C. Positive control treatment consisted of PVC tubes filled with non Cr VI^ +^ contaminated sand plus nematodes and one *G. mellonella.* Negative control treatment received Cr VI^ +^ contaminated sand but no nematode. Replication was 10-fold and the whole experiment was repeated twice in the same conditions but at different time period. Three days after incubation, insect death was recorded in each tube. Dead insects were dissected under a stereoscope in distilled water to confirm death caused by *S. feltiae*.

### 2.7 Data analysis

Experimental data were collected and inserted into an Excel file to constitute a database. That file was double checked for accuracy and curated for statistical analyses. The insect mortality in the pathogenicity experiment (section 2.2) was corrected with the insect mortality that occurred in the negative control using Abbott formula (% test mortality - % control mortality/ 100 - control mortality x 100) [24]. R software (version 4.1.2) and R Studio (version 2022.12.0 + 353 for Mac) were used to analyse the data and construct graphs. Initially, data were checked for normal distribution using the Shapiro-Wilk normality test. Thereafter, the Bartlett test was used to verify the homogeneity of variances. When normality and homogeneity of variances were not met, the non-parametric Kruskal Wallis test was run followed by a Post Hoc analysis to compare mean insect mortalities and number of progeny emerged after nematode reproduction inside the insect hosts. To assess the effect of Cr VI^ +^ on the development of the symbiotic bacteria, OD_600_ values were inserted into an Excel spreadsheet and mean values per treatment were calculated along with the standard deviations, and bacterial growth graphs were constructed using those values.

## 3. Results

### 3.1 Effect of Cr VI^ +^ on nematode pathogenicity to an insect host

**Fig 1:**
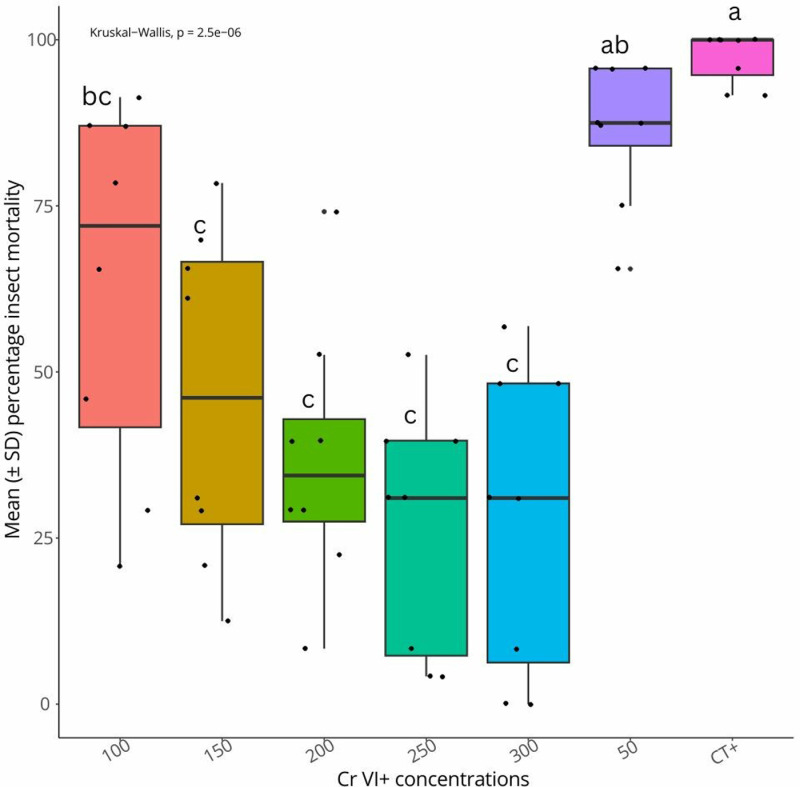
Mean ( ± SD) insect mortality percentages caused by *S. feltiae* in Cr VI^ +^ contaminated sand at various concentrations. Treatments with the same letter are not significantly different.

After three days incubation of the 24-well plate, dead *G. mellonella* larvae turned into black colour as indicated in S1 Fig, while the alive ones remained cream. Results showed that mean insect mortalities in plates varied significantly according to the Cr VI^ +^ concentrations (Chi square =  36.21454, df =  6, p-value < 2.504193e-06). The highest mortality (97.38%  ±  0.039) was recorded in the positive treatment CT + and lower insect mortalities (46.07% ±0.5 – 27.98% ±0.81) were recorded in 150-300ppm Cr VI^ +^ concentrations (Fig 1).

### 3.2 Effect of Cr VI^ +^ on nematode penetration percentage into an insect host and sex ratio

**Fig 2:**
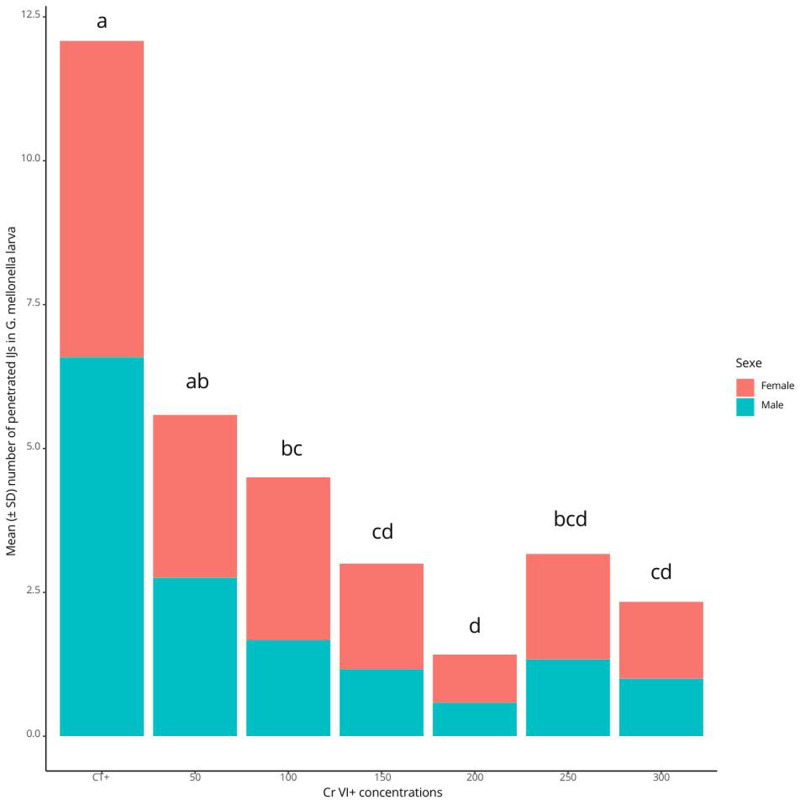
Mean ( ± SD) number of *S. feltiae* infective juvenile penetration in *G. mellonella* larvae in Cr VI + contaminated sand. Treatments with the same letter are not significantly different.

**Fig 3:**
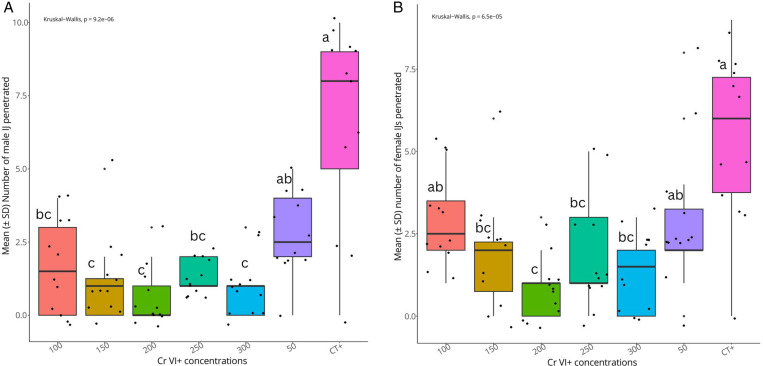
Sex ratio of *S. feltiae* infective juvenile penetration in *G. mellonella* larvae exposed to Cr VI ^ +^ contaminated sand. (**A)** represent the number of male IJ and (**B**) the number of female IJ that penetrated in *G. mellonella* in the presence of Cr VI^ + ^. Treatments with the same letter are not significantly different.

Results showed that the number of IJ (males and females) that penetrated the *G. mellonella* larva varied significantly among the Cr VI^ +^ concentrations tested (Chi square =  38.80, df = 6, P = 7.84001e-07). The highest number of penetrated IJ (12 ±  5 IJ) was recorded in the positive control CT + compared to the other Cr VI^ +^ concentrations tested (Fig 2). The numbers of female IJ (Chi square =  28.85, df = 6, P = 6.482872e-05) and male IJ (Chi square =  33.29, df = 6, P = 9.227733e-06) that penetrated the insect larvae were also significantly different among the Cr VI^ +^ concentrations tested. Higher numbers of penetrated male and female juveniles in insect host were recorded in the positive control treatment (Fig 3A and 3B).

### 3.3 Effect of Cr VI^ +^ on the reproduction potential of *S. feltiae*

**Fig 4:**
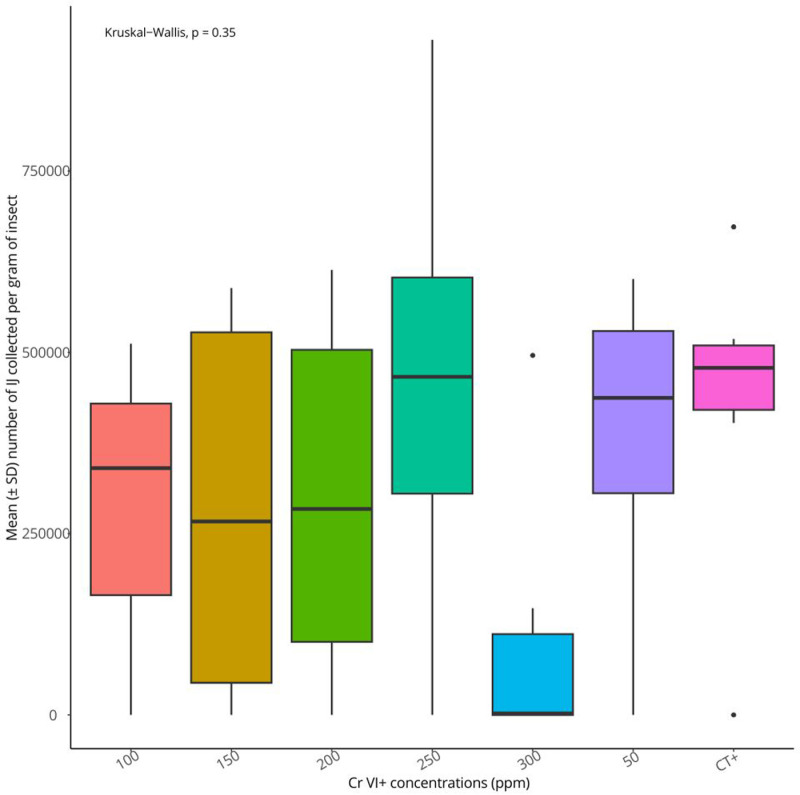
Reproduction of *S. feltiae* in *G. mellonella* in Cr VI^ +^ contaminated sand at various concentrations.

**Fig 5:**
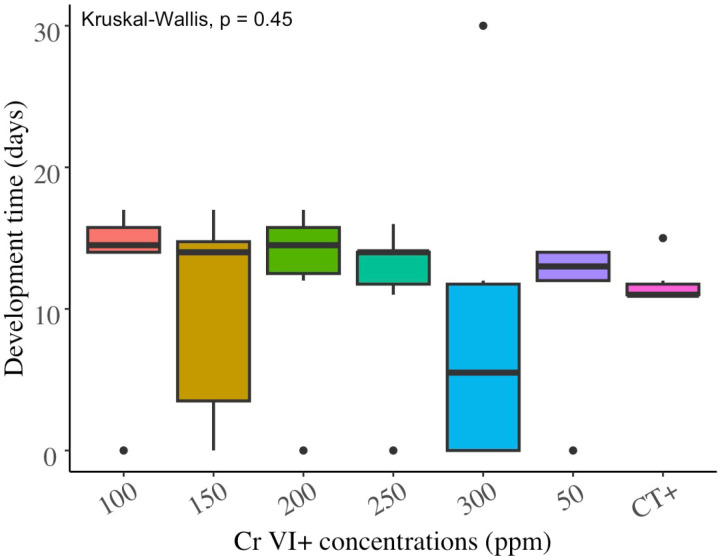
Development time (Mean ( ± SD) number of days) of *S. feltiae* in *G. mellonella* exposed to Cr VI^ +^ contaminated sand.

**Fig 6:**
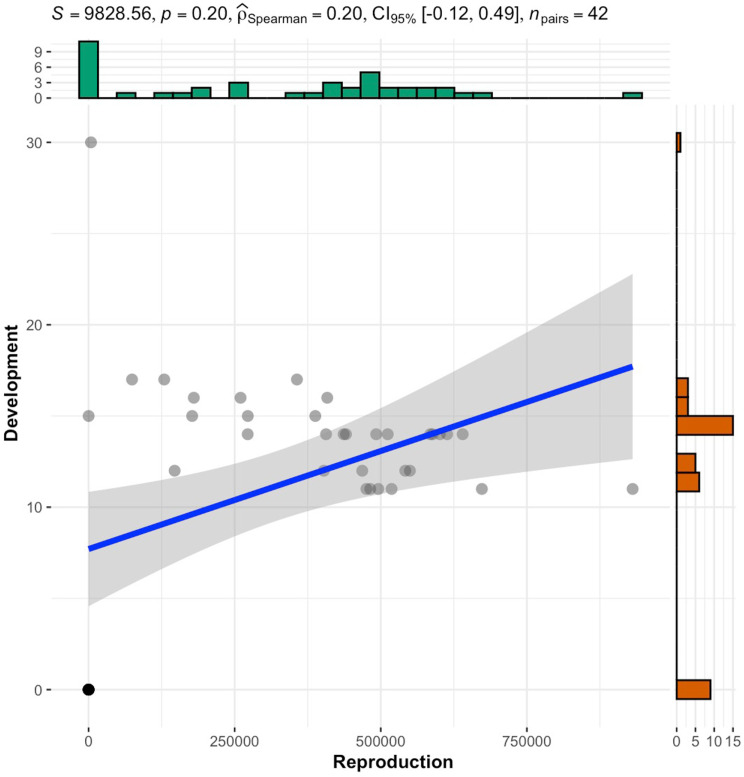
Spearman correlation between the development time (number of days elapsed from insect death and the emergence of progeny) and the reproduction of *S. feltiae* in *G. mellonella* exposed to Cr VI^ +^ contaminated sand.

Results showed that the numbers of *S. feltiae* progenies per gram (weight) of *G. mellonella* larva were not significantly different (Chi square =  6.727655, df = 6, P = 0.35) among the Cr VI^ +^ concentrations tested up to 300 ppm (Fig 4). The nematode development period, expressed in the number of days elapsed before the first IJ emerged in the White traps (Fig 5) appeared not to vary significantly among Cr VI^ +^ treatments as well (Chi square =  5.78095, df = 6, P = 0.45). A minimum period of 11 days was required to observe the first IJ in the White traps. Up to 478727 ±  92579 IJ/gram were recorded from a single *G. mellonella* larva. There was a weak positive correlation (Spearman correlation coefficient = 0.20) between the number of IJ emerged from *G. mellonella* cadavers and their development time inside the dead insect before emergence (Fig 6).

### 3.4 Effect of Cr VI^ +^ on the development of the symbiotic bacteria of *S. feltiae*

**Fig 7:**
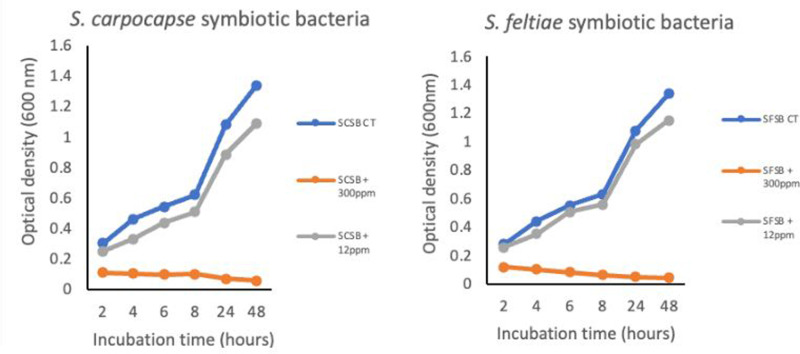
The effect of hexavalent chromium on the development of *S. feltiae* symbiotic bacteria in nutrient broth media. SFSB CT = Control treatment = *S. feltiae* symbiotic bacteria + Nutrient broth; SFSB +  300 ppm = *S. feltiae* symbiotic bacteria +  300 ppm Cr VI^ + ^; SFSB +  12 ppm =  *S. feltiae* symbiotic bacteria +  12 ppm Cr VI^ + ^; SBSB CT = Control treatment = *S. carpocapsae* symbiotic bacteria + Nutrient broth; SBSB +  300 ppm = *S. carpocapsae* symbiotic bacteria +  300 ppm Cr VI^ + ^; SBSB +  12 ppm =  *S. carpocapsae* symbiotic bacteria +  12 ppm Cr VI^ + ^.

On solid TSA and NBTA media, the development of the symbiotic bacteria of *S. feltiae* was not inhibited. Similar results was obtained with the positive control containing the symbiotic bacteria of *S. carpocapsae*. As shown in S1 File, the spots where the Cr VI^ +^ drops were added did not exhibit any prevention in the bacterial growth. Furthermore, the two tested bacteria were able to grow in liquid medium NB at low Cr VI^ +^ concentration (12 ppm), but no growth was observed in the same condition at 300 ppm (Fig 7).

### 3.5 Nematode attraction to the host in Cr VI^ +^ contaminated sand

**Fig 8:**
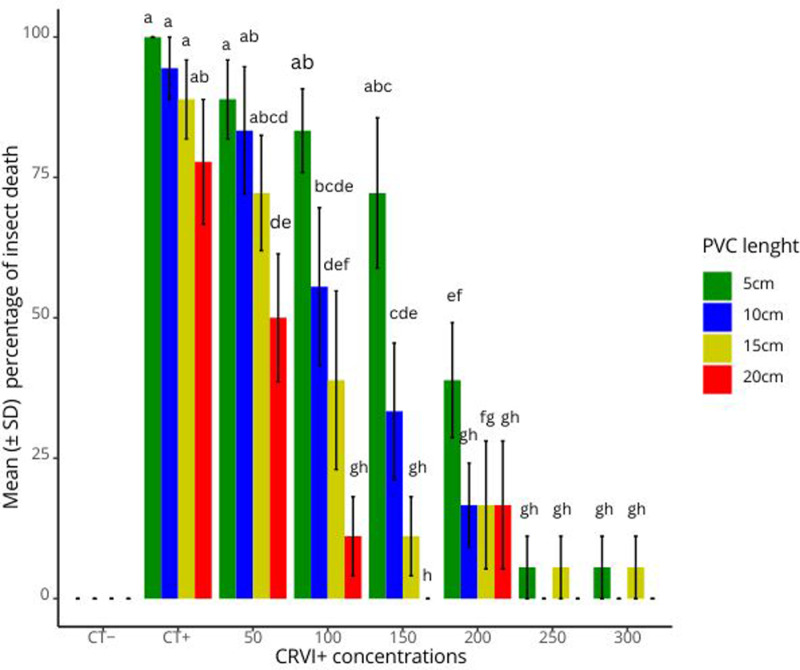
Percentage of insect mortality caused by *S. feltiae* nematode in PVC tubes of 0-20 cm length filled with varying concentrations of Cr VI ^ + ^. Treatments with the same letter are not significantly different.

Results showed that in Cr VI^ +^ contaminated sand (50-300 ppm), IJ were able to move through the PVC tubes of up to 15 cm length to kill *G. mellonella* larvae in all treatments (Fig 8). Insect mortality was also recorded in 20 cm tubes filled with 200 ppm Cr VI^ +^ contaminated sand. Nonparametric analysed revealed that mean insect mortality levels varied significantly among Cr VI^ +^ concentrations (Chi square =  124.24, df =  7, p-value < 2.2e-16) and PVC tube length (Chi square =  13.218, df =  3, p-value =  0.004188) treatments. Higher mean insect mortalities were recorded in the positive control (CT+) and 50ppm Cr VI^ +^ (Fig 8). Kruskal post hoc analyses showed that regardless of the PVC tube length, the mortality percentages recorded in the negative control (CT-) treatments were significantly different from the mortality percentages recorded in the positive control, and at 50 and 100 ppm Cr VI^ +^ concentrations. However, mortality percentages recorded in the 150-300 ppm treatments appeared to be similar to those in the negative control. Surprisingly, mortality levels recorded in the 150 ppm treatment and the CT + were similar (Table 1). An in depth pairwise analysis (S2 File), revealed that treatment 20 cm PVC * 150 ppm Cr VI^ +^ caused significantly different insect mortality than 5 cm PVC*CT + and 10 cm PVC * CT + treatments.

**Table 1:** Kruskal post hoc comparison of insect mortality levels among Cr VI^ +^ concentrations. Alpha: 0.05.

Comparisons (Cr VI + conc)	obs.dif	critical.dif	stat.signif
100-150	23.062500	50.10974	FALSE
100-200	29.333333	50.10974	FALSE
100-250	60.875000	50.10974	TRUE
100-300	60.875000	50.10974	TRUE
100-50	33.687500	50.10974	FALSE
100-CT-	66.250000	50.10974	TRUE
100-CT +	48.708333	50.10974	FALSE
150-200	6.270833	50.10974	FALSE
150-250	37.812500	50.10974	FALSE
150-300	37.812500	50.10974	FALSE
150-50	56.750000	50.10974	TRUE
150-CT-	43.187500	50.10974	FALSE
150-CT +	71.770833	50.10974	TRUE
200-250	31.541667	50.10974	FALSE
200-300	31.541667	50.10974	FALSE
200-50	63.020833	50.10974	TRUE
200-CT-	36.916667	50.10974	FALSE
200-CT +	78.041667	50.10974	TRUE
250-300	0.000000	50.10974	FALSE
250-50	94.562500	50.10974	TRUE
250-CT-	5.375000	50.10974	FALSE
250-CT +	109.583333	50.10974	TRUE
300-50	94.562500	50.10974	TRUE
300-CT-	5.375000	50.10974	FALSE
300-CT +	109.583333	50.10974	TRUE
50-CT-	99.937500	50.10974	TRUE
50-CT +	15.020833	50.10974	FALSE
CT--CT +	114.958333	50.10974	TRUE

Insect mortality comparison between any other treatments of the 150 ppm and the CT + were similar. When the PVC lengths were considered, the post hoc analyses revealed that significant differences in the mean insect mortality levels were only observed in the 20 cm tubes compared to the 5 cm ones. All the other treatments induced similar mortality levels (Table 2).

**Table 2:** Kruskal post hoc comparison between insect mortality rates among different PVC tube lengths tested. Alpha: 0.05.

Comparisons (PVC length)	obs.dif	critical.dif	stat.signif
10 cm-15 cm	6.40625	29.9261	FALSE
10 cm-20 cm	19.93750	29.9261	FALSE
10 cm-5 cm	17.42708	29.9261	FALSE
15 cm-20 cm	13.53125	29.9261	FALSE
15 cm-5 cm	23.83333	29.9261	FALSE
20 cm-5 cm	37.36458	29.9261	TRUE

## 4. Discussion

The biological effects of toxicants can be better understood by toxicity experiments that employ single species bioassays [25]. For over two decades, nematodes have been utilised as bioindicators of soil quality, and they have demonstrated a strong capacity to evaluate the effects of heavy metal contamination on soil [11]. In 2018, Boyle and Kakouli-Duarte [15] investigated the effect of Cr VI ^+ ^ on the pathogenicity of *S. feltiae* to *G. mellonella* at 0-100 ppm metal concentration, with 10 ppm increments. Their results indicated that the IJ infectivity on *G. mellonella* in Cr VI^ +^ contaminated soils was significantly reduced. This assumption is fully supported by the results presented here, which showed that at 0-300 ppm Cr VI^ + ^, the *G. mellonella* larvae mortality appeared to be significantly different among the various tested metal concentrations. In the negative treatment (CT + -), the *G. mellonella* larvae survived up to 300 ppm Cr VI^ +^ exposure, suggesting that death recorded in other treatments was mediated by the EPN. Positive control and lower concentrations of Cr VI^ +^ such as 50 ppm recorded the higher insect mortality percentages. This indicates that by increasing the concentration of Cr VI^ + ^, the infectivity of the nematode decreased even though the nematodes managed to cope well with the metal exposure. These results are also supported by Ropek and Gorczyca [26] who reported in their work a decrease in the infectivity of *S. feltiae* in soil polluted with cadmium, copper, lead and zinc. From 150 ppm to 300 ppm, lower insect mortalities were recorded, suggesting that the threshold of Cr VI^ +^ concentration from which the nematode infectivity is affected, lies above the 150 ppm concentration.

In the literature, most research works investigating the effect of heavy metals on nematode biology focus on studying nematode diversity and abundance in heavy metal contaminated environments [25,27,28]. In their study, Campos-Herrera et al. [27] reported that EPN total spatial distribution was not significantly affected by the presence of high levels of heavy metals. A low nematode abundance was observed using a qPCR-based identification method, whereas the conventional baiting method [29] employed in the same contaminated area failed to isolate a single EPN. One can speculate that the infectivity of the few nematodes that survived the high heavy metal contamination may have been impacted, preventing them from parasitising the insects in the baiting system. As advised by the authors themselves, more thorough soil sampling need to be carried out in the same area to clarify the situation.

Contrary to the hypothesis in the present study, results indicated that there were no significant differences between the mean number of progeny per unit of insect weight among the Cr VI^ +^ concentrations tested (0 – 300 ppm) as well as the development time. On one hand, these results are not in line with the findings of Monteiro et al [25] who studied the effect of different heavy metals, such as arsenic, cadmium, on *Caenorhabditis elegans* development and found that reproduction proved to be a much more sensitive endpoint than other parameters tested. Knowing that soil nematodes differ in their sensitivity to soil pollution [30], the results presented here could be explained by the fact that a different nematode species and a different heavy metal were investigated. Indeed, in a similar study, Jaworska and Tomasik [8] noted that the activity of *S. feltiae* and *H. bacteriophora* IJ was affected differently by several heavy metals. According to previous studies [24], *S. feltiae* may show some genetic flexibility in response to contaminants. In addition, Jiang et al. [31] concluded in their work that the “feeding” behavioural endpoint is the relatively ideal sublethal toxicity endpoint of heavy metals including chromium to *C. elegans,* compared to other physiological endpoints such as growth and reproduction.

On the other hand, previous research conducted by Boyle and Kakouli-Duarte [15] recorded varying *S. feltiae* reproduction potential when the latter was exposed to 0-100 ppm Cr VI^ +^ in increments of 10 ppm. A significant positive correlation between increasing concentrations of Cr VI^ +^ in soils and nematode offspring production was observed, suggesting that the presence of Cr VI^ +^ in the habitat of *S. feltiae* may have a favourable effect on population numbers. The authors reported that there was some inconsistency in the results. For example, 80 and 90 ppm (Cr VI + concentrations) appeared to produce significantly different numbers of progeny when compared to 10 ppm, but at 100 ppm exposures, the number of progeny was similar to 10 ppm. Furthermore, when the nematode development times inside *G. mellonella* before emergence were compared, 60 and 80 ppm showed significant different number of days than those at 10ppm, but no difference was recorded with development days in response to 70, 90 and 100 ppm. This noticeable inconsistency in the results is the reason for the increased Cr VI^ +^ concentration tested in this study to match the natural occurrence levels of the metal in Irish agricultural soil [6]. This was also done to be able to detect a clear cutoff point whereby the nematodes start expressing the effect of Cr VI^ +^ exposure. To achieve this, in the current study, a single *G. mellonella* larva was exposed to the same number of IJ throughout all treatments instead of replacing dead larvae with alive ones in the same experimental pot [15]. In addition, contrary to the Boyle and Kakouli-Duarte [15] approach, all results were included in the statistical analyses in the present study, even hosts in failed White traps. Rahoo et. al [32] reported that there was no significant relationship between the inoculation dose and the number of *S. feltiae* progeny, while invasion in insect hosts was significantly increased with higher nematode doses. Failure or success of nematode reproduction in an insect host can be due to many parameters, such as the number and sex of IJ that penetrated the insect host [32,33], the temperature [34], and the success of the symbiotic bacteria multiplication inside the insect cadavers [35,36].

In this study, the number of IJ (males and females) that penetrated the insect hosts was significantly different among the tested Cr VI^ +^ concentrations, suggesting that nematodes penetrated differently the insect hosts in response to the levels of metal contamination. In addition, higher number of females and males were recorded in the positive control. These results therefore suggest that IJ penetration is not a factor that could affect the uniformity in nematode reproduction arising from nematodes exposed to various Cr VI^ +^ concentrations. Higher female penetration in the insect host should, logically, yield a higher number of progeny, but several additional factors are involved in the success of EPN reproduction. Further study could evaluate the direct effect of Cr VI^ +^ on the biological behaviour of the adult stage of the nematode. Boyle and Kakouli-Duarte [16] attempted this by injecting the dead *G. mellonella* hosts with Cr VI^ +^ (400 ppm) and they observed the reproduction of the nematode *in vivo*. However, no data on sex ratio was reported and this could have been interesting to investigate.

While studying the effect of Cr VI^ +^ on the development and growth of the symbiotic bacteria, results revealed that in liquid medium (NB), the symbiotic bacteria were not able to multiply at 300 ppm Cr VI^ +^ concentration, but they could effortlessly grow at low concentration (12 ppm). When exposed to the same metal concentration in the nematode attraction to the host in Cr VI^ +^ contaminated sand (section 2.6), the nematodes were able to migrate and parasitise the insect host at varying distances (5 and 15 cm tube length). Furthermore, reproduction was observed from IJ exposed to 300 ppm Cr VI^ + ^. These results could be explained by the fact that the symbiotic bacteria, known to play a crucial role in the infection and reproduction process in the insect host [35], are naturally carried in the gut of the nematodes and were not exposed to high level of heavy metal due to a possible low bioaccumulation of the compound by the nematode. Currently, there is no clear information in the literature reporting the bioaccumulation potential of EPN to Cr VI^ + ^. This knowledge gap is currently being investigated by the Molecular Ecology and Nematode Research Group aiming to further understand the mechanism by which the nematode survives the presence of Cr VI^ +^ and continues its life cycle in a contaminated area.

On NBTA and TSA, *Xenorhabdus* did not exhibit any growth inhibition 48h hours post Cr VI^ +^ exposure at 300 ppm. The fact that the bacteria tolerate that high metal concentration on solid media, but not in NB, remains unclear. Further investigations focusing on studying the growth of the bacteria on several metal concentrations both in solid and liquid media could provide additional insights on this. Moreover, a slight delay in the bacterial growth (lower OD values) was observed in low metal concentration (12ppm Cr VI^ + ^; Fig 7) compared to that in the control treatments which received no Cr VI^ + ^. This observation (not statistically proven in this study), could be further investigated in future studies including more Cr VI^ +^ concentrations. This could potentially explain the delay in the nematode development time previously reported [15].

In the nematode attraction to the host in Cr VI^ +^ contaminated sand experiment (section 2.6), IJ of *S. feltiae* were able to survive exposure to Cr VI^ +^ up to 300 ppm and could migrate through the tubes to kill the insect host placed at the opposite end up to 20 cm away. Wu et al. [37] indicated in their study that the presence of hosts can considerably enhance the migration of IJ through similar soil columns seeking infection. The difference between the mean mortality of insect hosts between the 5 cm and the 20 cm distances indicated that the lower the migration distance, the easier for the nematode to parasitise the insect host. The results in this study agree with previous findings where the virulence of native Beninese EPN to control tephritid fruit flies, was assessed in the absence of heavy metal contamination [38]. Furthermore, Ferguson et al. [39] showed in their study that other EPN species such as *Steinernema carpocapsae* and *Heterorhabditis bacteriophora* were recovered at 20 cm and 35 cm, respectively. Nematodes have different behaviour strategies with EPN species classified as having a cruiser, ambusher or intermediate foraging tactics [40]. In previous studies, *S. feltiae* has been classified as a cruiser [32,41] and very good at finding distant hosts in sandy soils [34]. This assumption is supported by the results in the present study, which showed that the nematode can move through varying levels of Cr VI^ +^ contaminated sand to locate, kill and reproduce in the insect host.

A good bioindicator organism is expected to be abundant and widely distributed. EPN are globally present in soil, but in numerous standard surveys conducted around the world, the proportion of samples that test positive for EPN is comparatively low, at less than 35% [42,43]. In Ireland, by the means of the conventional *G. mellonella* baiting method, *S. feltiae* was recovered from soil at 7.1% [44] and at 2.5% to 3.2% from a sand dune system [45]. Several biotic and abiotic factors may prevent or delay the nematode activity for infection in the baiting method [46,47]. Understanding the fators affecting EPN distribution in soil is crucial to enhance the ecosystem service these nematodes offer. Therefore, in addition to ecotoxicology studies, the need to expand our knowledge on novel sampling approaches to access EPN with high recovery frequency is evident [46–48]. For instance, by utilising the quantitative polymerase chain reaction (qPCR) molecular tool, Campos-Herrera et al. [27] discovered sympatric distributions of EPN species and their low abundance in samples, where the insect baiting technique was unsuccessful. Furthermore, in recent studies, the combination of conventional sampling methods with molecular tools in field studies allowed the selection of main driving EPN occurrence worldwide with increasing accuracy [49]. Such methodology facilitates the use of EPN as ecological model organisms to select more sustainable agricultural practices and more importantly as markers of soil health [49].

Overall, bioindication of soil pollutants should be based on tolerant nematodes. In their study, Ekschmitt and Korthals [30] provided a list of nematode genera that may potentially serve as bioindicators based on their tolerance to soil contaminants. Nematodes from the genus *Rhabditis* were listed as tolerant organisms to several heavy metals, including chromium. The results reported here support their conclusion and confirm the choice of *S. feltiae* as a potential sentinel organism to risk assess Cr VI^ +^ in contaminated sand. Compared to the various sublethal endpoints that were investigated, the reproduction parameter did not appear to be a suitable endpoint to be used to risk assess soil polluted with Cr VI^ + ^. However, further investigations on the Cr VI^ +^ bioaccumulation potential in *S. feltiae* and mathematical modelling analyses are being undertaken to select the best endpoints to develop a protocol for soil heavy metal risk assessment using *S. feltiae* as an environmental bioindicator.

## Supporting information

S1 Fig Insect mortality caused by *S. feltiae* in Cr VI^ +^ contaminated sand (50-300 ppm) 3 days after the incubation of plates in the dark at 20 C. CT ^+^ represents the positive control that received nematodes but not Cr VI ^+^ while CT- and CT + - are the two negative controls with CT- receiving no nematode and no Cr VI ^+^ and CT ^+^ - receiving Cr VI ^+^ but no nematode. (TIF)

S1 File *Xenorhabdus* bacteria isolated from *S. feltiae* (A) and *S. carpocapsae* (B) and grown on NBTA and TSA media. On NBTA and TSA, bacterial growth 48hours after exposure to Cr VI ^+^ at 300ppm are presented for *S. feltiae* (E, F) and *S. carpocapsae* (C, D), respectively. (PDF)

S2 File Pairwise analysis comparing insect mortality levels among Cr VI ^+^ concentrations and PVC lengths. (XLSX)
