# Supplementary figures and images for: Effects of hexavalent chromium on the biology of Steinernema feltiae: evaluating sublethal endpoints for ecotoxicity testing
Source: PLoS One. 2025 Apr 1;20(4):e0320329. doi: 10.1371/journal.pone.0320329 (PMC11960951; doi:10.1371/journal.pone.0320329)

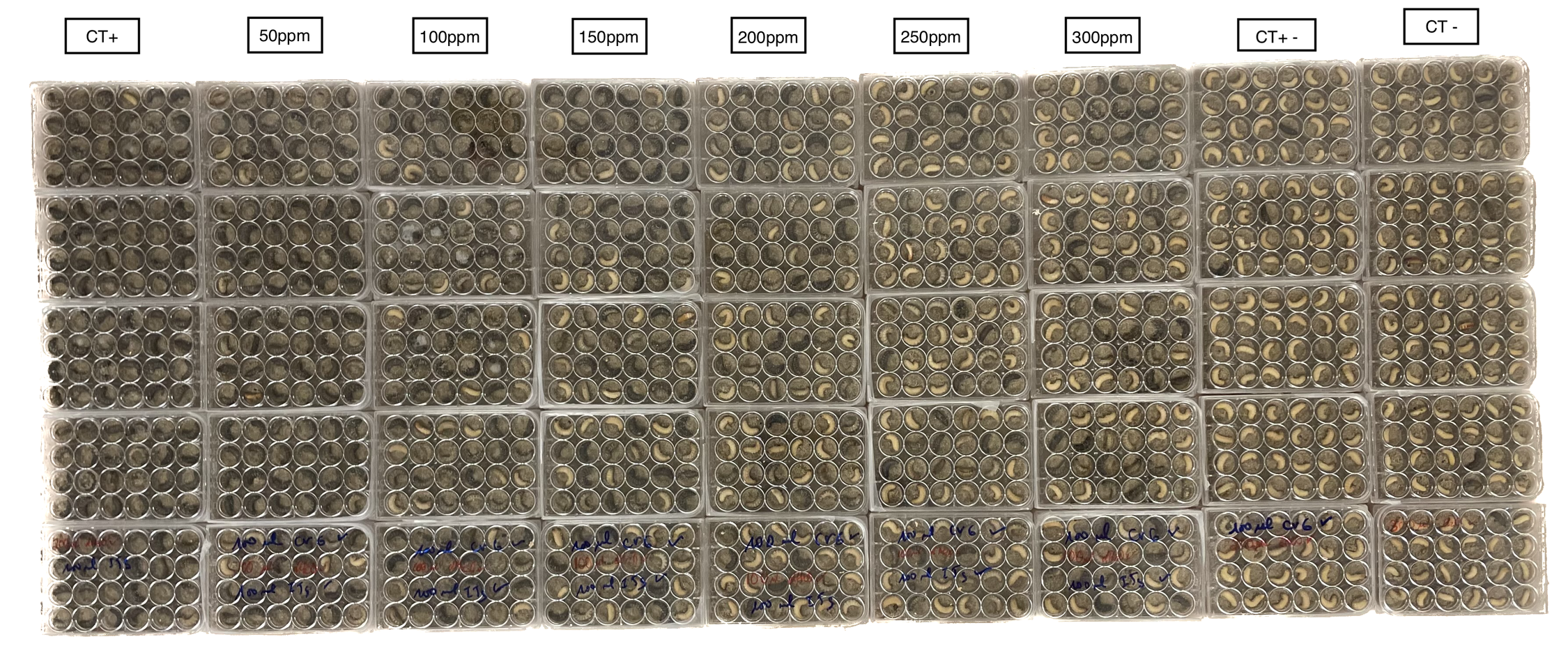

Supplement: S1 Fig — (TIF) [file pone.0320329.s001.tif]
