# Supplementary material for: Effects of hexavalent chromium on the biology of Steinernema feltiae: evaluating sublethal endpoints for ecotoxicity testing
Source: PLoS One. 2025 Apr 1;20(4):e0320329. doi: 10.1371/journal.pone.0320329 (PMC11960951; doi:10.1371/journal.pone.0320329)
Supplement: S1 File — (PDF) [file pone.0320329.s002.pdf]

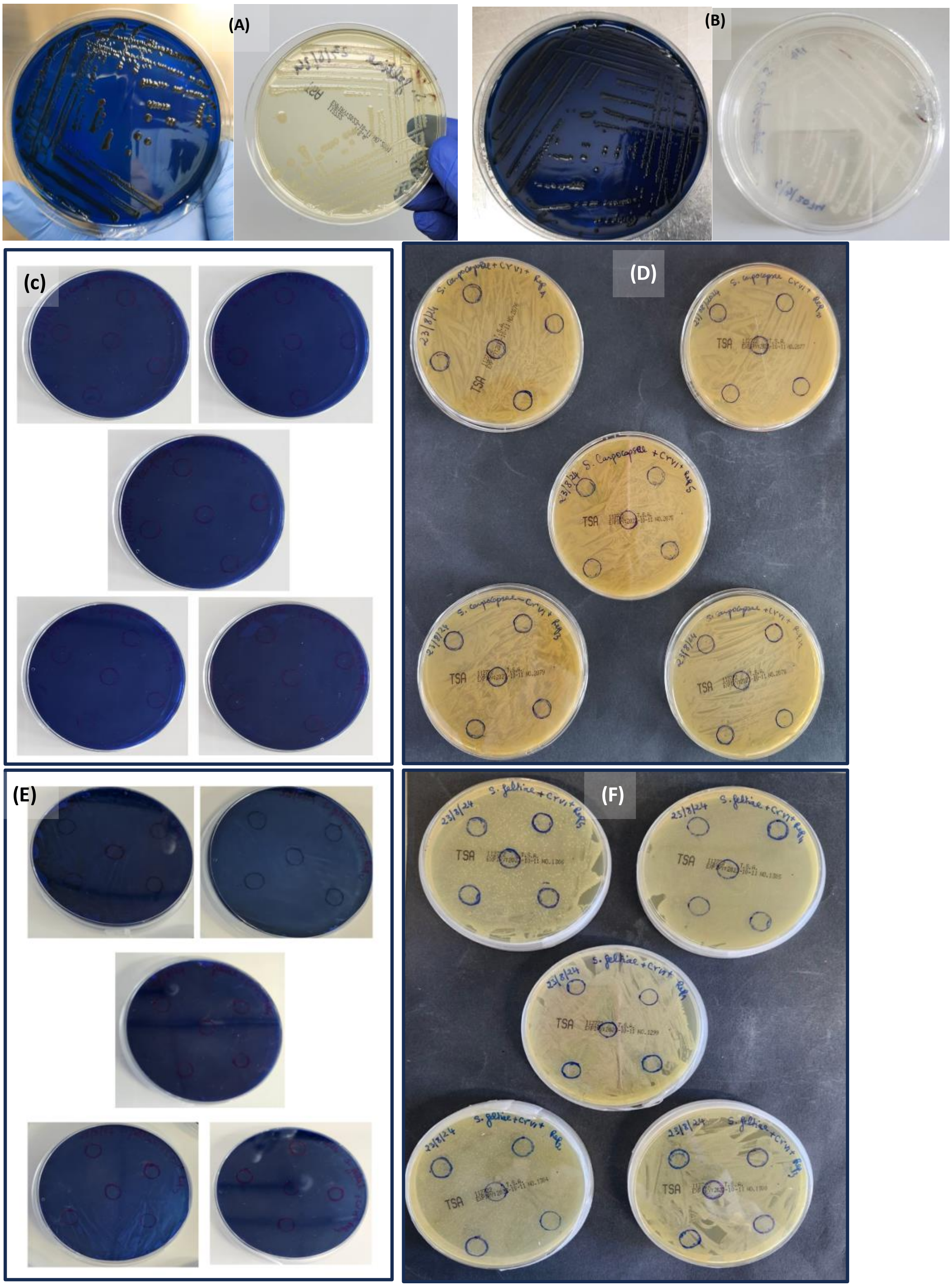

Supplementary material 2: *Xenorhabdus* bacteria isolated from *S. feltiae* (A) and *S. carpocapsae* (B) and grown on NBTA and TSA media. On NBTA and TSA, bacterial growth 48hours post exposure to Cr VI⁺ at 300ppm are presented for *S. feltiae* (E, F) and *S. carpocapsae* (C, D), respectively.
